# Indoor air formaldehyde (HCHO) pollution of urban coach cabins

**DOI:** 10.1038/s41598-019-57263-4

**Published:** 2020-01-15

**Authors:** Daocong Qin, Bing Guo, Jian Zhou, Heming Cheng, Xiaokai Chen

**Affiliations:** 1 College of Civil Engineering, Kunming University of Science and Technology, Kunming, 650500 China; 2 National Engineering Research Center of Waste Resource Recovery, Kunming University of Science and Technology, Kunming, 650093 China

**Keywords:** Environmental sciences, Civil engineering

## Abstract

Urban coach cabin is an important indoor environment for long journey, formaldehyde (HCHO) is a carcinogenic gas and damages indoor air quality of cabins. In order to control the HCHO pollution, the air samples inside cabins were analysed with a thermally desorbed gas chromatograph, and the HCHO diffusion was simulated with a methodology of computational fluid dynamics (CFD). Results show that through the experimental monitoring, the HCHO pollution level range from 33.6 to 142.3 μg/m^3^, decrease quickly with time, and the attenuation trendline is univariate cubic equation. Through the CFD simulation, the indoor temperature and HCHO level of cabin front and rear ends are higher than ones of other areas for the insufficient air supply and the unreasonable arrangement of air exhaust outlet. Moreover, through the CFD simulation, the HCHO level decreases with height growth of breathing zone and increasing air supply speed, and fresh air lead to diffusion of HCHO pollution from cabin seat area to the surrounding area. Through the CFD simulation, the HCHO pollution under the wind speeds of 3~5 m/s is higher than the HCHO limit level from indoor air standard of China vehicles, which shows that the HCHO emission of cabin seat has an important impact on airborne HCHO pollution inside vehicle cabins.

## Introduction

According to the year 2017 statistic bulletin of traffic industry development from the Transportation Ministry of China, the total of urban vehicles was 3.1 × 10^8^ and the annual capacity of passengers transported by the vehicles was 7.2 × 10^11^. For people often go working, shopping, traveling, school, home and lengthy commutes by driving or taking a vehicle such as a car, taxi, bus or coach, vehicle cabins have been recognized as important indoor environment, which result into indoor air quality (IAQ) and thermal comfort of vehicle cabins to be a hot research^1–8^. However, vehicle cabins have presented airborne formaldehyde (HCHO) and volatile organic compounds (VOCs) pollution, which damage vehicle IAQ^9–15^. For example, more than 260 VOCs^16^ were detected in the vehicle cabins and the maximum level of total VOCs was 6.90 mg/m^3^. Moreover, the HCHO pollution was relatively abundant in the coach cabins, and the HCHO highest level of 0.16 mg/m^3^ was observed on the 15th day after coming off the assembly line^11^. Except to pollute vehicle IAQ, the HCHO and VOCs pollution lead to the unacceptable health risks to passengers and drivers^17–19^. In order to decrease the health risks, China government promulgated the hygienic standard for air quality inside long distance coach (GB/T 17729–2009) and the guideline for air quality assessment of passenger car (GB/T 27630–2011), with the HCHO limit levels of 0.12 and 0.10 mg/m^3^ respectively. However, the average/maximal HCHO levels in car cabins were 132.0/251.6 μg/m^3^, which were 1.10/2.10 and 1.32/2.52 times more than the HCHO limit levels of IAQ standards on China coaches (GB/T 17729-2009) and passenger cars (GB/T 27630-2011) respectively^20^. The HCHO average level in taxi cabins was 1.0 ~ 1.4 mg/m^3^, which was 10 ~ 14 times higher than the HCHO limit level of China car IAQ standard (GB/T 27630-2011)^10^. So, there is a compelling need to initiate actions to reduce the HCHO pollution.

In order to control the HCHO pollution, the influencing factors of HCHO mass concentration were analyzed experimentally, and vehicle age, ventilation modes, cabin materials, air temperature and relative humidity did positively affect the HCHO concentration^9–11,20,21^. For example, when the cabin indoor temperature was from 29 °C to 35 °C, the HCHO level was from 100.6~115.7 to 172.8~251.6 μg/m^3^ respectively^20^. The HCHO emission quantity (*Q*)^9^ was generally related to cabin indoor temperature (*T*), and the logarithm of *Q* × *T*^−1/8^ was linear with *T*^−1^. Moreover, compared with no ventilation, the fan ventilation introducing outside fresh air decreased 49.5% of the HCHO level, and the recirculation ventilation without outside fresh air increased 51.38%^21^. Irrespective of engine and ventilation modes, the HCHO level inside cabins was significantly higher than one outside cabins, and the highest HCHO level was inside the mid-size car, followed inside compact car and large-size car^22^. In addition, the HCHO level in the vehicle cabin with leather trim was 1.42 times more than one in vehicle cabin with fabric trim^21^. Therefore, the experimental research shows that there are many factors affecting the HCHO pollution. In any case, the spatial distribution of HCHO pollution cannot be obtained by experimental monitoring, so the numerical simulation of HCHO pollution distribution needs to be analyzed.

Furthermore, the numerical simulation with computational fluid dynamics (CFD) has been used successfully to study the spatial and temporal distributions of cabin air temperature, air velocity and air pollution excluding HCHO^23,24^, which are technically difficult to measure in real vehicle conditions. For example, the particulate matter level was influenced by the outside air penetrated into vehicle cabins through the operation of cabin doors and ventilation system, and the CO_2_ level was elevated with increasing passengers in the vehicle cabin, which indicates that the cabin air pollutants were not diluted because of insufficient ventilation^24^. Through the CFD simulation, the eddies were generated in the mid upper region and each side of the vehicle cabins, which is indicative of the highly turbulent nature of cabin air flow^24^. Moreover, the ventilation modes, vent location and cabin seat arrangement affected the concentration distribution of cabin air pollution such as influenza, and the developed numerical model can provide insights into how the micro-environmental conditions affect cabin air pollution transmission^23^. However, there is few reports about the numerical simulation of airborne HCHO pollution in vehicle cabins, and the less is known about the effect of different breathing zone (horizontal plane), air temperature and ventilation conditions on the HCHO pollution. The coach is a main mean of transportation between different cities, and the time spent in the coach cabin is long enough to drivers and passengers. Therefore, this study is done in order to confirm the concentration distribution and control technology of cabin indoor HCHO pollution.

## Materials and Experimental Methods

### Coaches and roads investigated

Kunming City is the capital of Yunnan Province in the southwest of China, and locates in a mild climate zone with small annual temperature difference. Lijiang City locates in the northwest of Yunnan Province, and belongs to the monsoon climate of low latitude and warm temperate plateau mountainous areas. In China, coaches are the most commonly used means of transportations for long-distance travel, and the traffic on city roads is heavy during the tourism-peak reason. Moreover, in December 2018, this study was carried out, and the driving road from Kunming to Lijiang is 517 km long or 10 hour driving distance of coach. The coach sampled was made in China, and has been running in a regular service with air condition system, gasoline fuel, leather decoration and 42 seats. The coach age, exhaust volume and total driving mileage were 2.5 years, 3.6 liters and 5.9 × 10^4^ km respectively.

### HCHO Sampling and analysis

According to China standard, the detect methods for air quality inside long distance coach (GB/T 27380-2012), the HCHO pollution was sampled by the air sampler (QC-2) with dual gas flow path and was analyzed by the gas chromatograph (GC-6890) with a flame ionization detector, a 2 m capillary column and a desorption instrument. When the HCHO samplings is in process, the coach windows, doors and vents are closed and smoking is prohibited. Through the air sampler and the adsorption tubes with 150 mg adsorbent impregnated by dinitrophenylhydrazone, the HCHO samples was collected in the center at 0.5 m above coach floor with a 0.5 L/min flow rate for 50 L. The HCHO samples were desorbed by the thermal desorption instrument within 5 days of collection, were separated into fractions by the capillary column, and then were analyzed by the flame ionization detector. The temperature of column oven is programmed from 50 °C to 260 °C, the temperature of injection port and flame ionization detector are 230 °C and 260 °C, and the flow velocity of N_2_, H_2_ and air are 70, 40 and 450 mL/min, respectively. Moreover, around the coach cabins sampled, the atmospheric pressure was measured with a barometer (DYM3-1), the temperature and relative humidity were measured with a temperature-humidity recorder (AR-807), and all experimental instruments are made in China. Finally, the HCHO pollution are identified and quantified by comparing the HCHO samples curve to the corresponding HCHO standard curve. The calculation formula for HCHO level is as follows:

$$c=\frac{{B}_{s}\times {V}_{1}\times (h-{h}_{0})}{{V}_{0}\times {E}_{s}}$$

where: *c*, cabin indoor HCHO level, mg/m^3^;

*B*s, calculation factor from standard curve, μg/(mL·mm);

*E*s, average elution efficiency;

*V*_1_, total volume of eluent solution of sample, mL;

*V*_0_, sample volume in standard condition, L;

*h*, mean value of peak height of sample solution, mm;

*h*_0_, mean value of peak height of blank solution, mm;

### Quality control

Before the HCHO pollution sampling, the air sampler was calibrated by a soap film flow meter and all adsorption tubes were cleaned at least three times with high purity nitrogen. In the process of on-site sampling, two sampling tubes shall be left for non-sampling and treated as the blank sample. If the HCHO pollution in blank sample exceeds the HCHO limit level of national standard, all samples of on-site sampling shall be invalid. After sampling, the HCHO adsorption tubes were sealed with plastic end caps, placed in a sample box, and then transported to the GC-6890 laboratory. The detection limit of HCHO analyzing method is 0.01~1.0 mg/m^3^, and all HCHO sample concentrations are within the detection limit. Moreover, the HCHO standard solution, with purity of 98.5% and concentration of 1.0 mg/mL, is within the validity period and were obtained from the Institute for Reference Materials in Environmental Protection Ministry of China. The precisions of monitoring instrument for pressure, temperature and relative humidity are ± 0.1 kPa, ± 0.1 °C and ± 1.0%, respectively.

### Numerical simulation methods

The numerical simulation software is the ANSYS Software for Windows Version (19.0), which is composed of Space Claim Release 19.0 (SCDM 19.0), ICEM CFD Release 19.0, Fluent Release 19.0 including CFD-Post, and Tecplot 360 EX 2017 R3.

### Case geometry

**Figure 1:**
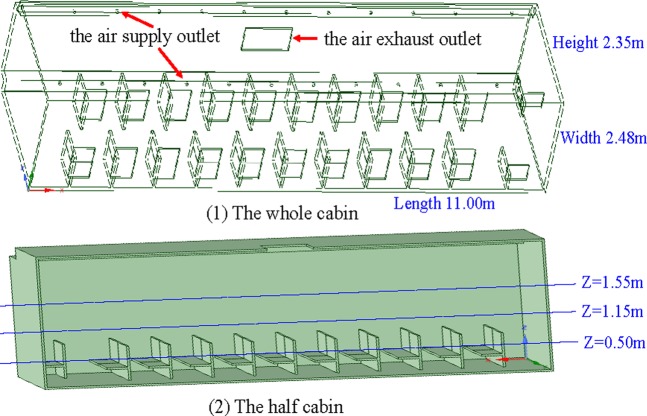
Geometry of coach cabin.

According to the actual size of coach cabin which length, width and height are 11.00, 2.48 and 2.35 m respectively, the cabin geometric model is constructed for numerical simulation. The cabin has 42 seats of length 0.50 m, width 0.40 m and height 0.90 m, the air supply outlet (eleven circles, φ = 0.06 m) is located on the left and right sides of cabin roof, and the air exhaust outlet (one rectangle, 1.0 m × 0.6 m) is arranged in the middle of cabin roof. The structure of coach real cabin is complex, and in order to facilitate the simulation calculation, the cabin model is simplified without doors and windows, as shown in Fig. 1 **(1)**. Because of the symmetry, the half of cabin model is used to numerical simulation in order to reduce computational load, as shown in Fig. 1 **(2)**.

### Mesh generation

**Figure 2:**
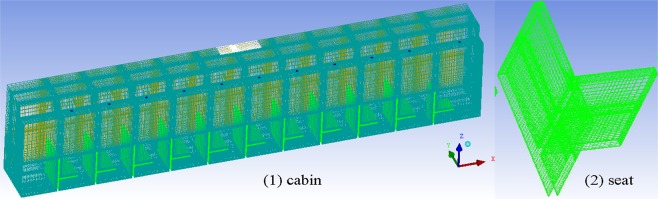
Schematics of the cabin and seat mesh.

The computational domain, coach cabin, is meshed with unstructured grids, and for the large gradient of physical parameters in flow field, the meshing of air exhaust outlet, air supply outlet and seat surface is appropriately refined to better satisfy the numerical simulation, as shown in Fig. 2. The grid total of computational domain is 2315420, the quality of best grid is 1.0, and the percentage of grid quality over 0.9 is more than 89%.

### Mathematical modeling and boundary conditions

Boundary conditions of coach cabins is complex so that some assumptions have been put forward. (1) The cabin walls do not emit HCHO, and is adiabatic; (2) The mixed air in cabins is ideal and incompressible; (3) The cabin seat is the only source of HCHO, the emission rate and diffusion coefficient of HCHO is constant; (4) The HCHO concentration of air supply is 0 mg/m^3^. Moreover, the physical model of cabin simulated is three-dimensional steady state, and the solution parameters include the Pressure-Based Coupling Solver, the SIMPLEC scheme for pressure-velocity coupling, the implicit difference equation, the absolute velocity equation. The control equation is energy equation, the turbulence equation is standard *k-ε* model with standard wall function, Turbulent Kinetic Energy is 1 m^2^/s^2^, Turbulent Dissipation Rate is 1 m^2^/s^3^, and the component equation is a general finite rate model, Species Transport. Cabin temperature and air velocity were monitored by the air velocity meter (Model 9565 series, made in TSI of America). The cabin indoor HCHO pollution is from the HCHO emission of cabin seat surface, and the other boundary conditions are shown in Table 1.

**Table 1:** Boundary conditions used in the numerical simulation.

Boundary types	Conditional parameters
Air supply opening	Velocity: 3, 4 and 5 m/s, Temperature: 18 °C Airflow rate: 0.09, 0.12 and 0.16 m^3^/s
Air exhaust opening Floor board	No-slip, Free outflow, Flow rate weighting: 1 No-slip, adiabatic
Other walls	No-slip, adiabatic, Temperature: 27 °C
Pollution emission source (seat)	Mass-Flow Inlet, HCHO diffusion coefficient: 2.88 × 10^−5^ HCHO emission rate: 7.4 × 10^−11^kg/s^34^

## Results

### HCHO concentration analysis

**Figure 3:**
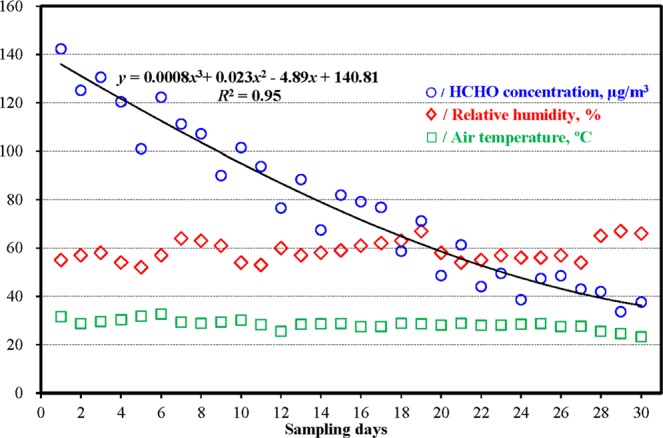
Scatter diagram of cabin air temperature, humidity and HCHO concentration.

The average, median, minimum and maximum of the HCHO concentration were 78.0, 76.7, 33.6 and 142.3 μg/m^3^ respectively, and the HCHO maximum were 1.19 and 1.42 times more than the HCHO limit levels of IAQ standards on China coaches (GB/T 17729-2009) and passenger cars (GB/T 27630-2011) respectively, which is attributed the HCHO emission of cabin leather materials, the HCHO release from food brought in and the insufficient ventilation. However, in Xiamen city of China^11^, in the cabins of medium-size coaches on the 15^th^ day after coming off vehicle assembly line, the HCHO mass concentrations were 0.16 mg/m^3^, which was higher than our monitoring result. The reason for the difference of HCHO concentration is the difference of coach brand, coach age, sampling location, cabin indoor temperature and relative humidity. Moreover, as shown in Fig. 3, the HCHO pollution decrease quickly with the growth of sampling days. After the 10, 20 and 30 days, the HCHO pollution decrease by 34.1%, 56.9% and 73.6%, respectively. For the HCHO and VOCs pollution mainly comes from cabin indoor materials including leather trims, organic solvents, paints, adhesives and so on^11–13,25^. In contrast with old vehicles, new vehicles have the newer materials with the stronger HCHO emissions, which lead to the higher HCHO pollution. When the HCHO flow rate from cabin inside to outside exceeds that from indoor materials into vehicle cabins, the HCHO pollution is more and more low. Through the curve fitting analysis of HCHO concentration (*y*, μg/m^3^) attenuation with sampling time (*x*, days), the attenuation trendline is univariate cubic equation, *y* = 0.0008*X*^3^ + 0.023*X*^2^ − 4.89*X* + 140.81 and *R*^2^ = 0.95. Furthermore, in Fig. 3, cabin air temperature is relatively stable for the cabin was sealed during the monitoring period, while cabin relative humidity shows some fluctuations for the impact of rain.

### Cabin air distribution in breathing zone

**Figure 4:**
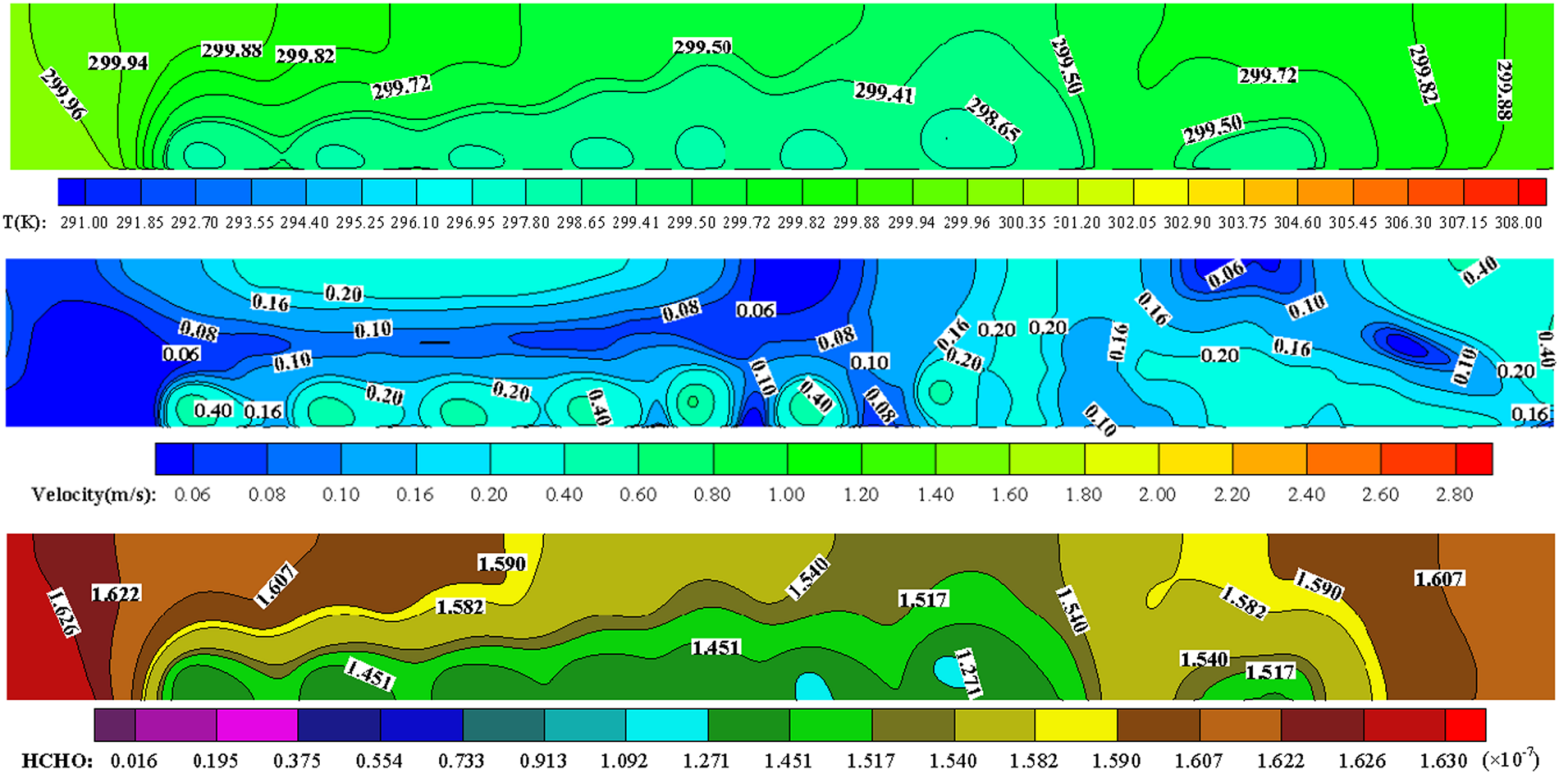
Spatial distribution of cabin air temperature, flow velocity and HCHO mass fraction in breathing zone of passengers sitting (Z = 1.15 m, V = 3 m/s, T = 18 °C).

As shown in Fig. 4, the air supply at cabin front and rear end is insufficient when the flow velocity of cabin air supply outlet is 3 m/s, so that the waste heat at both ends cannot be discharged from the air exhaust outlet in time, which result into that the temperature at cabin both ends is higher than that in cabin middle. Through simulation and conversion, the minimum, average and maximum temperatures in breathing plane (Z = 1.15 m) of passengers sitting are about 18 °C, 26 °C and 35 °C respectively. Moreover, the local airflow at cabin both ends produces vortices, which hinder the discharge of HCHO pollution so that the cabin indoor air is slightly polluted by HCHO, as shown in Fig. 4. So, the ventilation at cabin both ends should be strengthened to reduce waste heat accumulation and to decrease the HCHO pollution. Furthermore, in the seat area near cabin window under air supply port, the wind speed is higher than that in other areas, and the air supply can effectively eliminate waste heat. The cold jet from the seat area near cabin window has a great influence on cabin flow field, and the cold jet is sent vertically downward in a straight-line shape, which may produce a strong sense of cold wind and a discomfort to passengers sitting.

### Spatial distribution of HCHO pollution in different locations

**Figure 5:**
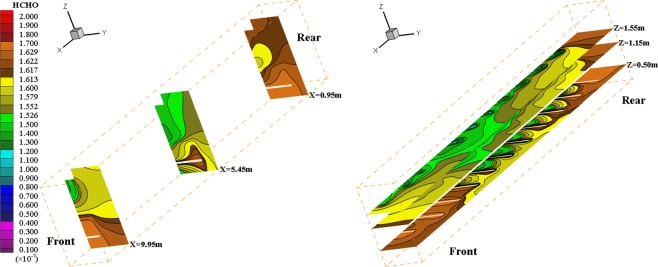
Spatial distribution of cabin HCHO mass fraction in different zone (V = 3 m/s, T = 18 °C).

As shown in Fig. 5, the HCHO concentration increases along the reduction of Z value, and the HCHO concentration in the lowest respiratory zone (Z = 0.50 m) is generally higher than other zones (Z = 1.15 m and Z = 1.55 m) for the lowest zone is close to cabin seat surface with larger emission area of HCHO pollution. However, with the growth of X value, the HCHO concentration first decreases and then increases for the air exhaust outlet is arranged in the middle and upper part of cabin. Moreover, the obstruction of cabin body wall leads to higher HCHO concentration in the upper area of cabin rear end, and fresh air dilutes the HCHO pollution around cabin window seats, which lead to the increasing HCHO concentration from the cabin seat area to the surrounding area. When the HCHO pollution diffuses near the air exhaust outlet, a part of HCHO pollution is discharged from the air exhaust outlet. Therefore, it is necessary to strengthen the ventilation design of cabin front and back ends and to increase the air supply volume of air conditioning, which can reduce the HCHO pollution.

### Spatial distribution of HCHO pollution in different wind speed

**Figure 6:**
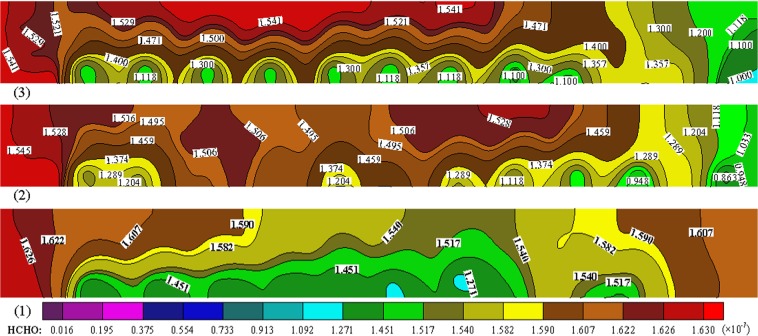
Spatial distribution of cabin HCHO mass fraction in breathing zone with different wind speed, Z = 1.15 m, T = 18 °C, (1) V = 3 m/s, (2) V = 4 m/s, (3) V = 5 m/s.

As shown in Fig. 6, the HCHO pollution decreases significantly with the increase of air supply speed, especially in the cabin rear area, which indicates that the increasing air supply speed is beneficial to reducing the HCHO level. The HCHO level in the seat area below air supply port is lower than that in other areas, and then increases gradually from the seat area to the aisle area because of the air exhaust outlet in the middle and upper part of cabin. In driver area, the HCHO level is heavy under the three kinds of wind speeds, which shows inadequate ventilation in the driving area and the unreasonable design of air exhaust outlet. By conversion, when the wind speed is 3, 4 and 5 m/s, the average concentration of HCHO pollution is 0.178, 0.159 and 0.158 mg/m^3^ respectively which exceed the maximum limit level of vehicle IAQ standard (GB/T 17729-2009 and GB/T 27630-2011) from China. Therefore, the HCHO emission of cabin seat has an important impact on vehicle indoor HCHO level, and cabin seat materials should be cleaned to control airborne HCHO pollution in vehicle cabins.

## Discussion

In general, the HCHO pollution decline quickly with the sampling days. In fact, the emission of HCHO pollution would gone through three periods such as accelerated emission (↗), stable emission (→), rapid decline (↘), and the HCHO emission rates from cabin indoor materials usually decrease with the sampling days. For example, the HCHO pollution exhibited an approximately inverted U-shaped pattern with car age of the 0^th^, 15^th^, and 30^th^ day after coming off the assembly line, which shows that the HCHO pollution increases from the 0^th^ to 15^th^ days and declines from the 15^th^ and 30^th^ days^11^. However, in the current research, the vehicle age was 2.5 years when the HCHO pollution was sampled, and the HCHO emission passed the front two periods (↗, →) and entered the rapid decline periods (↘). Moreover, cabin ventilation can decrease the HCHO pollution and old vehicles have the longer time ventilation than new vehicles, which lead to that the HCHO pollution declines with the sampling days. Furthermore, through the CFD numerical simulation, ventilation can reduce cabin indoor temperature and HCHO pollution, and in the poor ventilation of cabin front and rear ends, waste heat and HCHO pollution cannot be discharged in time, resulting in higher cabin indoor temperature and HCHO concentration, respectively. For windows, doors, solar radiation, lighting heat, human body heat, engine heat dissipation and other HCHO pollution sources are not considered, it is a certain difference between the simulation results and the actual results. The mass concentration of HCHO pollution was 142.3 μg/m^3^ on the first day of sampling. On the location of actual monitoring HCHO pollution, the HCHO level through the CFD simulation was 135.6 μg/m^3^ which was lower than the actual monitoring level. In fact, cabin indoor HCHO pollution may come from cabin outdoor air and emissions of cabin indoor materials and components such as the moldings, upholstery, roof linings, carpeting, seats, curtains, floor boards, lubricants, foam, plastic, acrylonitrile butadiene styrene resin, leathers and fibers, and the organic solvents, paints and adhesives. In order to facilitate the simulation calculation, the cabin model is simplified and the seat is assumed to be the only source of HCHO. Therefore, the simulation result is lower than the measured result, and the CFD simulation is reasonable to some extent.

Although the simulation results show that the HCHO emission of cabin seats has an important influence on cabin indoor air HCHO pollution, the HCHO emission behaviors from indoor materials are relatively complicated. For example, except time, the HCHO steady state can be influenced by other factors including indoor temperature, relative humidity, solar radiation and cabin ventilation, and these factors may simultaneously affect the HCHO emission, which make that the physical mechanism analysis of HCHO emission is complex^20^. Moreover, the HCHO emission process of indoor materials is characterized by three key parameters including diffusion coefficient (DC), partition coefficient (PC) and initial emission concentration (IEC)^26–30^. For a certain value of the IEC or DC, the relative sensitivity coefficient decays rapidly over time and tends to a small value when the emission time is long enough^26^. Furthermore, the IEC directly determine the actual behaviors of HCHO emission, and there is a coupling relationship between the IEC and PC^27,31^. Both the IEC and DC raise with increasing temperature, while the IEC raises but the DC decreases with increasing relative humidity^26,27^. According to the material structural parameters, the HCHO properties and the environmental conditions, some theoretical prediction models have been developed to determine DC^32,33^. In addition, the HCHO emission ratios from indoor materials are unstable, which influence the HCHO pollution.

## Conclusions

Generally, cabin temperature, breathing zone height and ventilation conditions have great influence on airborne HCHO pollution in vehicles, and the HCHO pollution would decline with time when the HCHO diffusion rate from cabin indoor air to cabin outside exceeds the HCHO emission rate from cabin indoor materials into cabin indoor air. In order to reduce vehicle indoor HCHO pollution, ventilation of cabin front and rear ends, especially in driver area, should be strengthened and cabin exhaust outlet should be designed more reasonably. The current results provide some worthy references for green design of vehicle cabin ventilation, air conditioning system and cabin indoor material, and for reasonable division of cabin environment and spatial layout, which can reduce cabin HCHO pollution, improve vehicle IAQ and thermal comfort to ensure sustainable development of urban public transport. However, it is difficult to analyze entirely the HCHO pollution in real vehicle cabins due to a lot of influencing factors such as cabin temperature, relative humidity, air pressure, cabin materials, driving routes, passenger number, cabin ventilation, climate situations, and so on. Further research in the future is needed to fully understand the interaction of these influencing factors, and to analyze the dynamic theory of HCHO emission from cabin seats.
